# *Diplolaimelloides woaabi* sp. n. (Nematoda: Monhysteridae): A Novel Species of Free-Living Nematode from the Great Salt Lake, Utah

**DOI:** 10.2478/jofnem-2025-0048

**Published:** 2025-11-07

**Authors:** Julie Jung, Thomas R. Murray, Morgan C. Marcue, Thomas Powers, Solinus Farrer, Abigail Borgmeier, Byron J. Adams, Jonathan A. Wang, Gustavo Fonseca, Michael S. Werner

**Affiliations:** University of Utah, School of Biological Sciences, Salt Lake City, UT 84112; Weber State University, Department of Zoology, Ogden, UT 84403; University of Nebraska-Lincoln, Department of Plant Pathology, Lincoln, NE 68583-0722; Brigham Young University, Department of Biology, Provo, UT 84602; Monte L. Bean Museum, Brigham Young University, Provo, UT 84602; Federal University of São Paulo, Marine Science Institute, Santos, São Paulo 04021-001, Brazil

**Keywords:** biodiversity, extremophile, microbialite, Monhysteridae, nematode, taxonomy

## Abstract

A new species of free-living nematode inhabiting microbialites in Great Salt Lake, Utah, USA is described both molecularly by 18S-sequencing and morphologically with scanning electron microscopy and differential interference contrast (DIC) microscopy. *Diplolaimelloides woaabi* sp. nov. (family Monhysteridae, order Monhysterida) is characterized by a combination of the following characters: ocelli present; a relatively small body size (<1.5 mm); short anterior sensory setae; cryptospiral amphidial fovea; a funnel-shaped anterior buccal cavity and reduced secondary cavity; fused lips; long double spicules and conspicuous male bursa displaying four pairs of post-cloacal papillae arranged in a (2 + 2) pattern, a single mid-ventral pre-cloacal papilla, two pairs of papillae posterior to the bursa, and an additional offset mid-tail papillae pair; and a pair of sub-apical extensions on spicules. An updated key to all species of *Diplolaimelloides* Meyl, 1954 is given. This species is notable for its adaptation to hypersaline microbialites, positioning it as both an extremophile and a potential bioindicator of ecological change in Great Salt Lake.

The family Monhysteridae de Man, 1876 is one of the most abundant and widely distributed nematode groups. They are characterized by being small (usually <1.5 mm length) and slender with finely striated body cuticles that are visible in scanning electron microscopy (SEM), and one elongated gonad along the right side. Genus and species-level designations within Monhysteridae have been revised several times (Gerlach, 1951; Timm, 1966; Jacobs, 1987; Eyualem et al., 2006; Fonseca and Decraemer, 2008; de Ward and Russo, 2009; Chen et al., 2023), but an increasing emphasis on describing male characters is steadily improving the systematics. The current taxonomy includes 18 genera which inhabit a diversity of marine, brackish, and limnic ecosystems (Fonseca and Decraemer, 2008; Abebe et al., 2012) and several extreme aquatic environments including saline lakes (Freckman and Virginia, 1997; Borgonie et al., 2011; Shih et al., 2019; Hodda and Traunspurger, 2021).

Monysteridae is the primary free-living nematode family inhabiting the south arm of Great Salt Lake (GSL) (Jung et al., 2024a) (Fig. 1A). Cursory morphological observations and SSU-genotyping indicated a major bottleneck, wherein a cosmopolitan community in a freshwater-river input effectively collapsed to a single family (Monhysteridae) in GSL (Jung et al., 2024a, 2024b). In addition to brine flies (*Ephydra sp*.) and brine shrimp (*Artemia* sp.), GSL nematodes currently represent only the third metazoan taxa found within the hypersaline bays of GSL. Previous sampling efforts revealed that nematodes are recovered in far greater numbers from “microbialites,” lithified mounds built by microbes, and may use them as a bacteria-rich *refugia* in this extreme environment (Fig. 1B).

**Figure 1:**
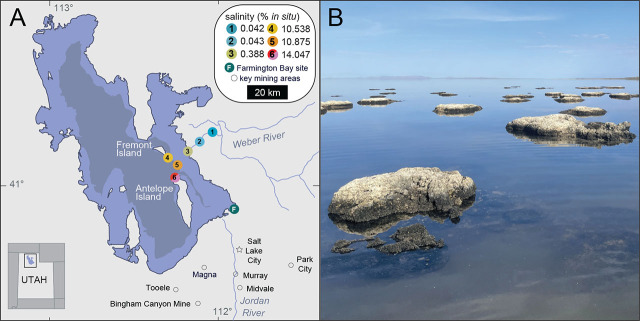
Sampling sites within the GSL. (A) Locations and average *in situ* water salinity of six sample sites around (1–3) and within (4–6) the GSL. Lake topographies depict NASA SRTM2 v.2 data from 2007 and UGRC LiDAR data from 2016. Figure adapted from Jung et al. (2024a, 2024b). (B) Representative microbialite mounds at Site 6 from which nematodes were extracted. GSL, Great Salt Lake.

The discovery of nematodes in GSL has potential ecological significance. Nematodes are known bioindicators of environmental disturbance, and their presence, abundance, and morphology can reflect shifts in water quality, salinity, or sediment chemistry (Danovaro et al., 2009; Hodda and Traunspurger, 2021). When considering the ongoing anthropogenic pressures on GSL (Naftz et al., 2008; Wurtsbaugh et al., 2016; Null and Wurtsbaugh, 2020), this species could serve as a valuable sentinel for monitoring ecological change. Moreover, its restriction to microbialites hints at specialized ecological interactions, possibly including microbial symbioses or unique life-history traits that warrant further investigation. Given the importance of microbialites for primary production in GSL, any ecological interactions of nematodes with other organisms, either positively or negatively, will affect the overall ecosystem of GSL.

Here, we use light microscopy with DIC and SEM to provide morphological descriptions of GSL nematodes. DIC images were combined with specimen-matched 18S-genotyping. Examination of both datasets reveals that nematodes from GSL represent a new species within the genus *Diplolaimelloides* Meyl, 1954 (Figs. 2 and 3). We present the description and diagnosis of *Diplolaimelloides woaabi* sp. nov. below and provide an updated species key, adhering to modern guidelines for species descriptions of free-living aquatic nematodes (Mokievsky et al., 2024).

**Figure 2:**
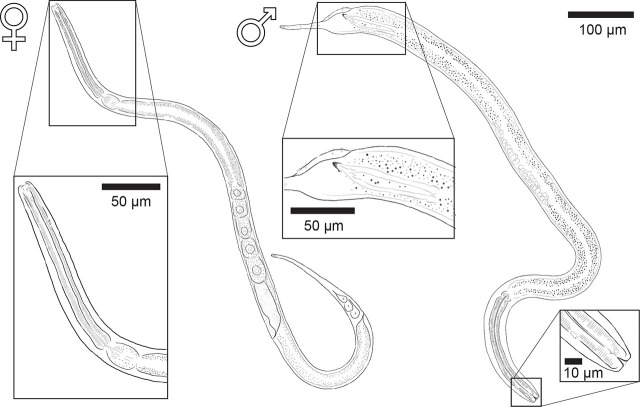
*Diplolaimelloides woaabi* sp. nov. Left: holotype female with anterior end inset. Right: holotype male with anterior and posterior end inset, to scale.

**Figure 3:**
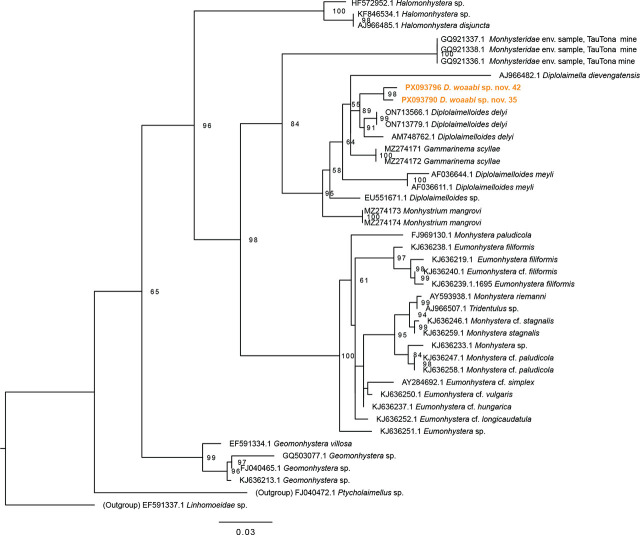
Maximum-likelihood phylogenetic tree of Monhysteridae nematodes, based on aligned SSU sequences, including two paratype male specimen of *D. woaabi* sp, nov. indicated in orange. Alignment made with MUSCLE and tree made with IQ-TREE (see section “Materials and Methods”). Branches with less than 50 bootstrap support are not reported.

## Materials and Methods

### Study site and sampling

Sediment samples of microbialites (top 10 cm of submerged microbialite mounds) were collected using a shovel from Bridger Bay, on the northern tip of Antelope Island within Great Salt Lake, UT, USA (41°03′32.4″N 112°15′28.8″W, salinity: 115 ppt, elevation 4,200 ft) from May to October of 2024 (Fig. 1). Nematodes were extracted from sediment samples either by sucrose density centrifugation or by placing a small <2 cm “nugget” of microbialite into a 50 ml conical tube with lake water, lightly shaking the tube, pouring the supernatant onto a Petri dish, and visually scanning the sample for live nematodes with a dissecting microscope. Isolated nematodes were further identified by microscopy and SSU-sequencing.

### Morphological characterization

Light microscopy/DIC observations and measurements were primarily made on live specimens, anesthetized using 100 mM sodium azide on 2% agarose slides under a differential interference contrast microscope (DIC, Leica DM2500). Mosaics and line drawings (Fig. 2) were made in Adobe Illustrator (v28.7.3) by digitally tracing the superimposition of multi-focal plane light images. All measurements were obtained using ImageJ (v2.14.0/1.54f) and all curved structures were measured along the arc or median line, presented in micrometers (µm) (Table 1). Unless explicitly mentioned, measurements provided in the text and tables are from DIC images of nematodes extracted from the wild; *n* = 21 females and 10 males.

### Scanning electron microscopy preparation

Living nematodes were initially fixed in 4% cold gluteraldehyde for 24 hr and rinsed in 0.1 M sodium cacodylate buffer, before fixation in 2% Osmium tetroxide for 8–12 hr. Nematodes were again rinsed in cacodylate buffer, followed by dehydration in a progressively-increasing concentration of cold ETOH solutions until the nematodes were in 100% ETOH. Nematodes were critical point dried, coated with silver, and mounted on a SEM stub before viewing on a Hitachi S4700 field-emission scanning electron microscope at the Morrison Microscopy Core Research Facility of the Nebraska Center for Biotechnology or Brigham Young University Electron Microscopy Laboratory.

Type specimens (in both glass vials and permanent slides) were fixed and deposited in the Utah Natural History Museum in Salt Lake City, Utah, USA. For glass vials, nematodes were extracted from 100 g of sediment sample by sucrose density centrifugation, heat killed in a water bath at 75°C for 12–15 min, and preserved in equal volume of hot 10% formalin. For permanent slides, nematodes were fixed as in glass vials and select nematodes were mounted on glass slides with a drop of glycerol and cover slides lifted with slim fibers of glass and sealed with nail polish.

### Molecular phylogenetic analysis

Individual worm lysate was prepared for two males, each submerged in 9.8 µl of single-worm lysis solution (121 mg Tris, 380 mg KCL, 51 mg MgCl_2_ × 6H_2_O, 460 µl NP-40, 460 µl Tween-20, fill to 100 ml ddH_2_O) and 0.2 µl of 10 mg/ml Proteinase K. Samples were heated for 2 hr at 65°C and 10 min at 95°C. Next, a primer pair targeting the 18S ribosomal small subunit (SSU rDNA) was used for PCR amplification; Forward primer SSU18A/RH5401: 5′–AAAGATTAAGCCATGCATG–3′; Reverse primer SSU26R/RH5402: 5′–CATTCTTGGCAAATGCTTTCG–3′ (Blaxter et al., 1998; Floyd et al., 2002). The amplicon length was approximately 1,000 bp. 500 bp was Sanger sequenced using the RH5403 primer: 5′–AGCTGGAATTACCGCGGC–3′ (Zhou et al., 2019) at MCLAB (CA, USA). Chromatograms were trimmed for quality in SnapGene Viewer (Snapgene software [www.snapgene.com]), and aligned with MUSCLE in AliView (v1.30) (Larsson, 2014) alongside other Monhysteridae 18S sequences from 18S-NemaBase (Gattoni et al., 2023) and recently described Monhysteridae genera that are found in the gill chambers of crustaceans, which are phylogenetically close to *Diplolaimelloides* (Westerman et al., 2022). *Linhomoeidae* sp. and *Ptycholaimellus* sp. were included as outgroups (Genbanks IDs are indicated in branch tips). The ~500 nucleotide block including GSL nematode sequences was used for realignment and saved as a .FASTA file, which was used to make a tree in IQ-Tree (v.3.0.1) (Nguyen et al., 2015). The general time reversible (GTR) model was used for nucleotide substitutions, and nodal support for maximum-likelihood analysis was performed with 1,000 bootstrap replicates (options: -st DNA -m GTR -bb 1,000 -alrt 1,000). The consensus tree was visualized in FigTree (v1.4.4) (Rambaut, 2018), and rooted on *Linhomoeidae* sp. Bootstrap values are indicated at each node, except for nodes with less than 0.50 support.

### Description

#### Systematics

Order MONHYSTERIDA Filipjev, 1929
Superfamily MONHYSTEROIDEA de Man, 1876
Family MONHYSTERIDAE de Man, 1876
Subfamily MONHYSTERINAE
Tribe MONHYSTERINI
Genus *Diplolaimelloides* Meyl 1954
*Diplolaimelloides woaabi* sp. nov.
(Fig. 2; Table 1).

**Table 1:** Measurements (µm) of *Diplolaimelloides woaabi* sp. nov. (range, mean value in parentheses).

	**Holotype female**	**Paratype female (*N* = 20)**	**Holotype male**	**Paratype male (*N* = 9)**
L	1,059.4	441.0–1,159.4 (712.5)	1,011.0	559.6–1,100.3 (835.5)
a	33.8	25.5–34.0 (28.8)	23.8	24.3–37.7 (31.9)
b	6.7	4.0–7.4 (5.7)	5.6	5.4–10.7 (6.6)
c	6.5	3.6–7.2 (4.8)	10.2	5.4–17.3 (10.0)
Stl	5.2	1.5–7.0 (3.5)	5.8	2.9–5.2 (4.0)
Stw	2.9	1.2–4.3 (2.2)	4.7	2.3–2.9 (2.6)
Bdcs	11.9	4.5–11.0 (7.1)	13.7	6.2–11.2 (7.8)
Csla	n/a	0.6–0.8 (0.7)	n/a	0.3–0.3 (0.3)
Aw	3.4	1.3–4.2 (2.5)	2.3	3.4–3.6 (3.5)
Al	3.5	1.3–4.2 (2.6)	2.3	3.4–3.6 (3.5)
Daa	14.4	10.8–20.0 (15.9)	17.8	13.8–15.6 (14.7)
A%	23.9	10.0–31.4 (16.5)	13	22.0–26.2 (24.1)
Bda	17.6	8.8–19.5 (11.8)	10.9	13.6–17.3 (15.5)
Ol	3.8	2.1–3.5 (3.1)	5.3	2.3–4.3 (2.9)
Ow	1.9	2.1–3.5 (2.5)	3.6	1.4–2.7 (2.1)
Dao	38.7	17.6–33.4 (25.5)	29.3	25.4–33.4 (29.0)
Bdo	20.1	13.5–19.9 (17.1)	25.4	1.2–18.7 (15.1)
Bdph	26.2	15.3–29.8 (20.6)	35.3	19.3–25.4 (23.0)
Daph	156.9	102.0–167.6 (126.3)	181	102.9–161.7 (136.0)
Mbd	31.4	17.0–35.5 (24.7)	42.4	22.6–31.7 (26.1)
Abd	23.3	15.0–28.2 (17.9)	34.3	17.1–28.7 (22.4)
Tl	164.2	87.8–210.1 (155.5)	99.3	53.4–122.7 (87.7)
Vbd	30.1	19.2–31.3 (23.8)	n/a	n/a
Vta	236.9	125.8–285.5 (165.2)	n/a	n/a
V%	62.1	49.8–61.8 (54.1)	n/a	n/a
B	n/a	n/a	54.5	22.1–52.3 (37.3)
S	n/a	n/a	89.5	57.1–77.0 (66.3)
Lfilt	134.4	29.5–139.4 (87.2)	70.4	26.6–87.0 (64.2)
Ltip	6.1	3.8–12.0 (5.9)	8.1	3.6–6.6 (5.0)

Abbreviations: Abd, anal/cloacal body diameter; Aw, amphidial fovea width; Al, amphidial fovea length; A%, amphidial diameter (average of length and width) as a percentage of body diameter; Bdcs, body diameter at level of cephalic setae; Bda, body diameter at level of the amphid; Bdph, body diameter at the level of pharyngeal end; Bdo, body diameter at level of ocelli; B, bursa length along the arc; Csl, cephalic setae length; Daa, distance from anterior end to amphid; Dao, distance from anterior end to ocelli; Daph, distance from anterior end to pharyngeal end; L, total length; Lfilt, length of filiform part of the tail; Ltip, length of transparent tail tip; Mbd, maximum body diameter; Ol, ocelli diameter from anterior to posterior; Ow, ocelli diameter from side to side; Pb, pattern of papillae on bursa, number of pairs/on one side only; Ppc, pattern of pre-cloacal papillae; Pcd, pattern of caudal papillae; S, spicule length along the arc; Stl, stoma length; Stw, stoma width; Tl, tail length or distance from anus/cloacal opening to posterior end; Vbd, body diameter at position of vulva; Vta, distance from vulva opening to anus; V%, distance from anterior end to vulva opening in percentage of total length; De Man’s ratios, a, b, and c used in this paper are calculated as standard.

### Adult specimens (sex independent)

**Figure 4:**
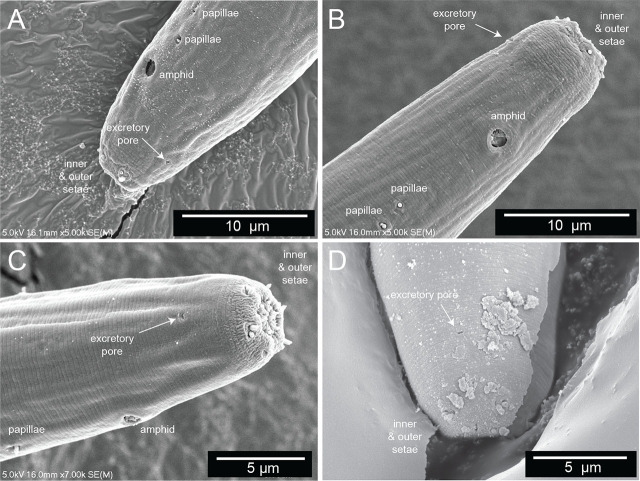
The secretory–excretory pore. (A–D) Scanning electron microscopy of the anterior end of four different *Diplolaimelloides woaabi* sp. nov. individuals. The position of the secretory–excretory pore on the ventral side of the nematode is annotated with a white arrow. Other miscellaneous salient features are labeled as landmarks.

**Figure 5:**
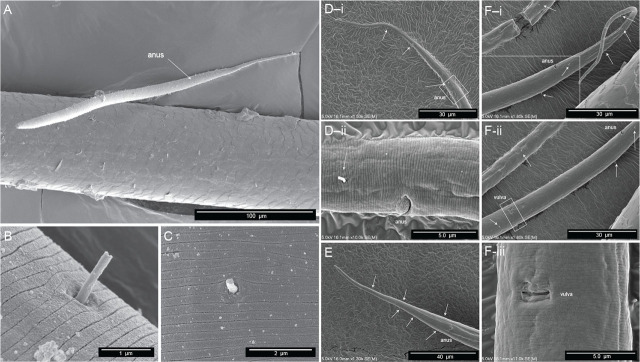
Scanning electron microscopy of female *Diplolaimelloides woaabi* sp. nov. shows several features, including the posterior pattern of papillae on (A) the whole body, (B,C) a closeup of integument and single papilla, and (D–F) the posterior ends of nematodes, with each letter depicting a different individual and successively zoomed-in shots of the anus and/or vulva areas with visible papillae labeled with white arrows.

**Figure 6:**
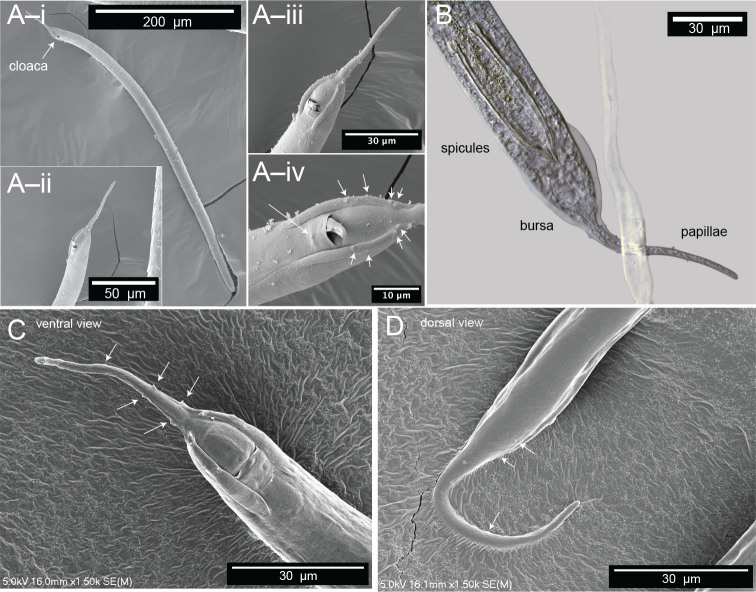
Microscopy of male *Diplolaimelloides woaabi* sp. nov. shows the posterior pattern of papillae on the whole body or posterior ends (A–D). Each letter depicts a different individual and successively zoomed-in shots with papillae labeled with white arrows. (B) Image taken with DIC microscopy; all others taken with scanning electron microscopy. DIC, differential interference contrast.

The body plan is radially symmetrical and nearly cylindrical, almost uniform in diameter, but tapered toward both ends, more so posteriorly at the tail (Fig. 2). The body is slender, with a diameter at the pharyngeal end at 20.9 µm in females and 24.6 in males. The ventral side can be recognized by the position of the secretory–excretory pore (Fig. 4) and the vulva and anus in females (Fig. 5) or the cloacal opening in males (Fig. 6). The cuticle appears thin (c.0.7 µm) and smooth in light microscopy but ornamented with subtle transverse striae in SEM (c.0.2 µm). No longitudinal markings are observed. The integument features a series of dorsal, lateral, and ventral papillae. The outer parts feature setae that vary from short (min length 0.2 µm) to long (max length 0.9 µm) (Figs. 5B,C).

**Figure 7:**
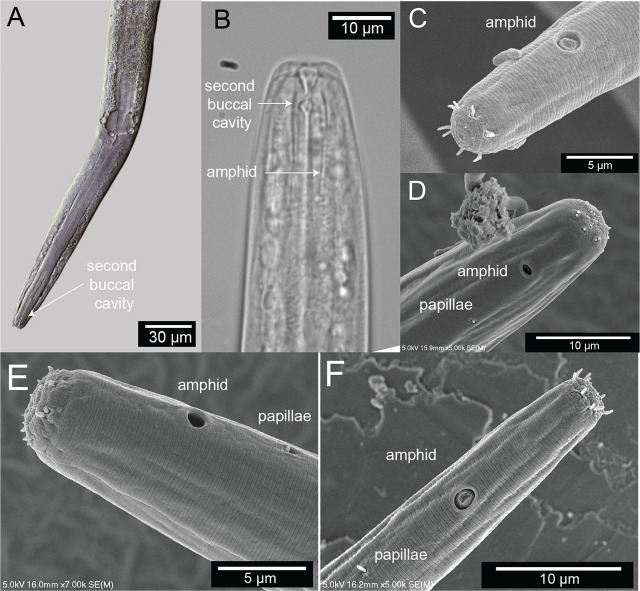
Terminal head region of *Diplolaimelloides woaabi* sp. nov. (A) DIC microscopy reveals stoma with a double cavity, or two distinct compartments, the muscular pharynx and the prominent cardia. (B) Section of the anterior end, arrows pointing to the second chamber of the buccal cavity and the position of the amphidal fovea. Scanning electron microscopy shows (C–F) completely fused lip plates that carry a concentration of sense organs in a 6 (inner) + 6 + 4 (outer) pattern, and amphids and somatic papillae visible slightly posterior from the anterior end. DIC, differential interference contrast.

The lip region is continuous with the body contour but slightly tapered rather than fully cylindrical. The oral opening is terminal and leads to a double cavity with a slight constriction in the middle portion dividing the cavity into two compartments (Figs. 7A,B). No denticles observed in the anterior buccal cavity, which is funnel shaped with cuticularized walls, relatively deep and narrow (Table 1). The basic pattern of the anterior region consists of six peri-oral plates radially arranged around the terminal oral opening: two subdorsal, two subventral, and two lateral. The lips appear completely fused such that the anterior end of the nematode shows a subtle hexagonal, compressed region (Figs. 7C,D). The head carries a concentration of anterior sense organs in two circlets of sensilla (sensory papillae) which surround the stoma opening (Figs. 7C,D). The inner ring is made of six inner labial sensilla surrounding the mouth, which are papilliform and seem to vary in how protruding they are from the head (~1 µm long) (Figs. 7C,D). The sensilla in the outer ring are made of six outer labial setae and four cephalic setiform sensilla, with the positions of the two “missing” lateral setae in the outer circle in line with a posterior pair of amphids (Figs. 7C,D). The sensilla of the outer ring tend to be longer (2–3 µm long, one-quarter to one-third of labial diameter) than those in the inner ring (Figs. 7C,D).

The pharynx is cylindrical, gradually enlarging in a posterior portion with a subtle but notable bulb (Figs. 2; 7A,B). The cardia is distinct between the pharynx and the intestine.

The amphids are positioned laterally and posterior to the stoma, in the pharyngeal region of the body (Figs. 7B–D). The anterior margin of the amphid lies at an average of 15.8 (female) and 15.7 (male) µm from the anterior body end (range 10.8–20.0 µm). In size, amphids are on average 17.0% (female) and 20.4% (male) of the corresponding body diameter (range 10.0%–31.4%). Amphidial fovea range from circular to cryptospiral (Figs. 7B–F). One or two papillae are visible just posterior (range 3.1–11.6 µm, mean 7.6 µm) from the amphid (Figs. 7D–F).

**Figure 8:**
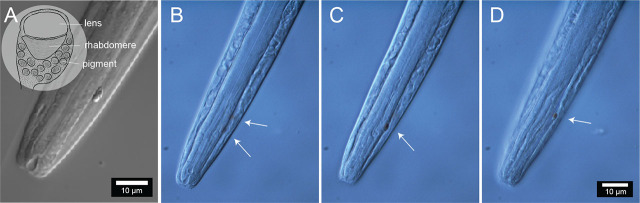
Photoreceptors of *Diplolaimelloides woaabi* sp. nov. (A) Anterior body end showing the position of one of the ocelli and detail of the pigment area. Inset shows a schematic reconstruction based on the ultrastructure of an ocellus, adapted from a TEM photograph (Van De Velde and Coomans, 1988). Anterior body end of a different specimen, showing the positions and colored pigment of (B) both, (C) one, and (D) the other ocelli.

Ocelli are present in males and females, located on average 28.1 (female) and 29.1 (male) µm from the anterior end (range 17.6–38.7 µm). The pigment associated with these structures is, for the most part, concentrated laterally or partly embedded in the pharynx (Fig. 8).

Ventral gland not easily observed in GSL nematodes using SEM or DIC microscopy. The three cell bodies of the caudal gland were observed in some but not all specimens. Caudal glands were associated with a spinneret, the terminal pore that helps secrete a sticky substance. Adherence to substrate material from the tail by isolated nematodes was observed, demonstrating that the spinneret is functional.

#### Females

**Figure 9:**
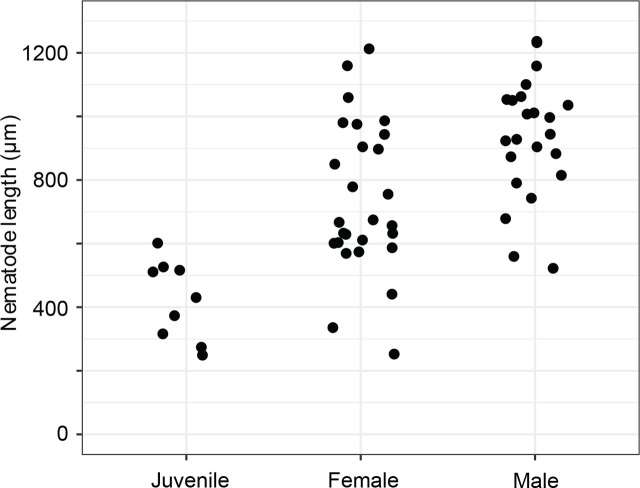
*Diplolaimelloides woaabi* sp. nov. total length as a factor of stage and sex.

**Figure 10:**
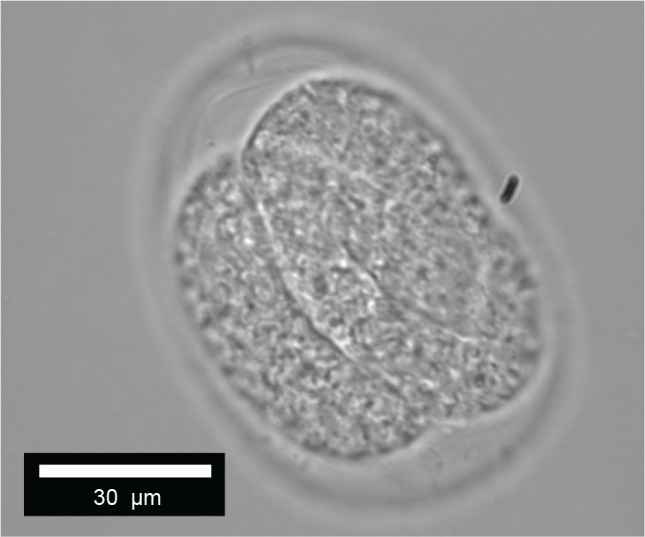
*Diplolaimelloides woaabi* sp. nov. egg.

The vulva position in females is variable but approximately halfway along the body (mean%V = 54.7%, range 49.8%–62.1%). The tail shape appears similar in all juvenile stages but shows strong sexual dimorphism between sexes as adults (Fig. 2). Females taper gradually from the vulva and the anus to the tail region for 12.7% of their length on average. The body lengths of female adults is also variable, between 441.0 µm and 1,159.4 µm (Table 1, Fig. 9). Females exhibit at least six caudal papillae in a 2 + 1 + 2 + 1 pattern; the first post-anal pair is sub-ventral and all posterior to that pair are lateral (Figs. 2; 5E). Eggs (mean length 52.1 µm, width 37.9 µm) were observed on agar plates post isolation of nematodes (Fig. 10).

#### Males

**Figure 11:**
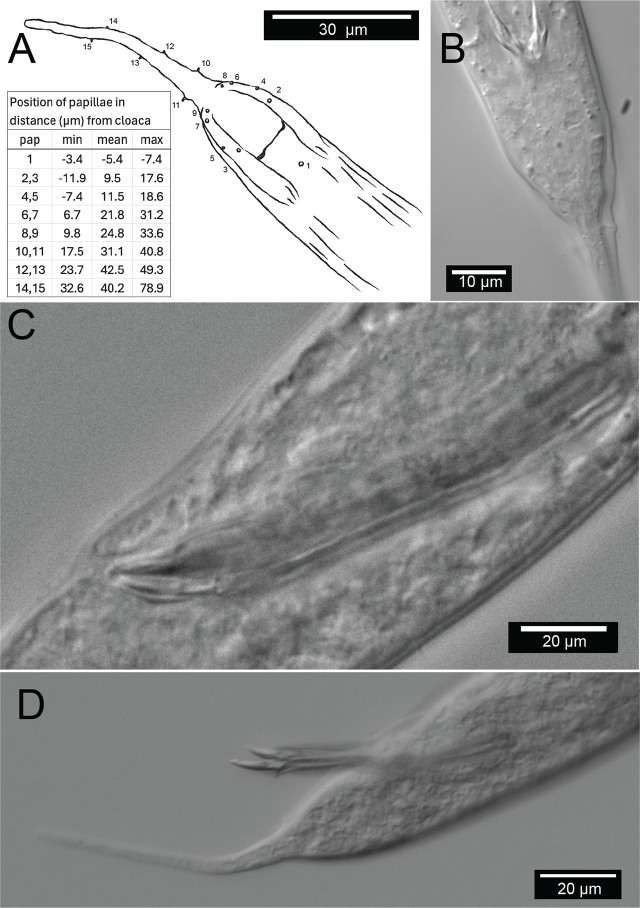
Male copulatory apparatus, *Diplolaimelloides woaabi* sp. nov. (A) Diagram of male papillae pattern and (inset) position. Gubernaculum in (B) ventral and (C) lateral view with non-emergent spicule. (D) Lateral view with emergent spicule. Images taken with or traced over DIC microscopy. DIC, differential interference contrast.

Males are relatively uncommon (~1%) in the population. From a dissecting microscope, the most obvious distinguishing characteristic of males is a body shape that narrows abruptly in the tail region, filiform for 8.1% of their length on average. Moreover, males present as slightly larger than females (Table 1, Fig. 9). The papillae pattern differs between females (Fig. 5) and males (Fig. 6). One single genital papilla can be found on the mid-ventral pre-cloacal surface on average 5.4 µm from cloacal opening of males (Figs. 6A,C). The male tail is enveloped by a conspicuous bursa (Fig. 6). The pattern of genital papillae associated with bursa are as follows: four papillae on each side arranged in an arc with a larger gap between two pairs of two (2 + 2) and all posterior to the cloacal opening (Figs. 6A,C). Three pairs of caudal papillae present posterior to bursa (Figs. 6B–D), including one pair of small “mid-tail” lateral papillae that can be seen in the ventral or dorsal view in males (Figs. 6B–D). The mean distance from each papilla to the caudal opening is shown in Figure 11A. The spicule is long and slightly arcuate, with ventral:dorsal extensions on either side below the tip, while the gubernaculum is short and funnel-shaped without apophysis (Figs. 11B–D).

#### Family and genus diagnosis

*D. woaabi* sp. nov. is characterized by their small size (typically <1 mm), slender with one elongated gonad on the right side of the intestine. Anterior setae are arranged in two circles of 6 + 10. A smooth cuticle with fine lateral lines are visible by SEM. The above features place *D. woaabi* sp. nov. in the family Monhysteridae. The presence of the bipartite buccal cavity (Figs. 7A,B), caudal bursa in males (Fig. 6), gubernaculum without dorsal apophysis, and long spicules (Fig. 11) place them within the genus *Diplolaimelloides*.

#### Species diagnosis

*D. woaabi* sp. nov. exhibits two ocelli. The amphids are circular, with a mean distance from the anterior at 15.8 µm in females and 15.7 µm in males. Sexual dimorphism of tail lengths include males with shorter tails than females and larger De Man’s c ratios (10 µm in males and 4.8 µm in females, Table 1). Long curved spicules and funnel-shaped gubernaculum without apophysis are seen. Spicules with ventral:dorsal extensions on either side resemble a “halberd” (Figs. 6A; 11B–D). One mid-ventral pre-cloacal papilla is located at an average of 5.4 µm from the cloaca in males. Caudal bursa exhibit four pairs of papillae in a (2 + 2) arrangement (first pair 10.2 µm and 15.4 µm from cloaca, second pair 23.1 µm and 25.4 µm from cloaca). *D. woaabi* sp. nov. also exhibit three post-bursal pairs: one at the base of the bursa, one offset pair more posterior but relatively anterior on the filiform part of the tail, and an additional, small, offset lateral pair approximately in the middle of the filiform part of the tail (Figs. 6B–D; 11A). Occasionally we observed small papillae near the proximal tip of the male tail, but it was too invariant or hard to see to be considered diagnostic.

In 2009, Pastor De Ward and Lo Russo introduced a species key to *Diplolaimelloides* focusing on adult male characters, that is, bursal papillae and morphology of gubernaculum and spicule (de Ward and Russo, 2009). These have shown to be useful diagnostic characters for subsequent descriptions of *D. rushikondai* and *D. contortus* (Sufyan et al., 2014; Chen et al., 2023). We adopted the most recent species key from Chen et al. (2023), which continues in this vein but also includes the presence/absence of ocelli (Chen et al., 2023). After working through the diagnostic key, we also provide additional differences between *D. woaabi* sp. nov. and the closest species (Table 2).

**Table 2.** Measurements (µm) of *Diplolaimelloides woaabi* sp. nov. (range) compared with measurements of all other described *Diplolaimelloides* species.

	***D. woaabi***		***D. altherii***		***D. brucei***		***D. conctortus***		***D. deconincki***		***D. delyi***		***D. elegans***		***D. islandicus***		***D. meyli***		***D. oschei***		***D. palustris***		***D. rushikondai***		***D. tehuelchus***		***D. warwicki***	
**Ref**	**Herein**		**(Meyl, 1954)**		**(Hopper, 1970)**		**(Chen et al., 2023)**		**(Gerlach, 1951)**		**(Andrássy, 1958, Chen et al., 2023)**		**(Gagarin and Thanh, 2008)**		**(De Coninick, 1943)**		**(Timm, 1961)**		**(Meyl, 1954)**		**(Tsalolikhin, 1985)**		**(Sufyan et al., 2014)**		**(Pastor de Ward & Lo Russo, 2009)**		**(Pastor de Ward and Lo Russo, 2009)**	
**Sex**	♀	♂	♀	♂	♀	♂	♀	♂	♀	♂	♀	♂	♀	♂	♀	♂	♀	♂	♀	♂	♀	♂	♀	♂	♀	♂	♀	♂
L	441–1159	560–1100	616–988	508–832	690–880	730–890	737–854	713–844	736	860	810–1410	690–1310	809–826	835	680	717–730	750–1200	820–1250	554–850	587–717	776–851	824	620–748	545–683	760–880	600–700	1000–1010	770–1100
a	25.5–34.0	23.8–37.7	33.0–40.0	37.7–42.2	41.9	36.9	23.0–29.5	25.9–32.5	21.2	24.8	26.3–40.2	27.3–44.5	28.0–30.0	35	30.2	42.7–43.7	25.7–37.9	31.4–43.5	33.0–41.7	34.8–37.0	27.4–31.0	34.3	26.0–37.0	32.1–42.7	33.0–35.0	32.9–35.0	35.7–40.4	30.8–35.5
b	4.0–7.4	5.4–10.7	6.5–7.7	5.5–6.4	7.0	6.5	6.6–7.1	6.1–7.0	6.8	6.3	6.2–8.9	5.8–7.9	7.0–7.4	7.2	5.6	5.2–5.4	5.1–6.7	5.0–6.5	6.0–7.1	6.0–6.8	6.6–6.7	6.2	6.8–8.0	6.0–7.6	5.7–6.3	5.6–6.2	7.4–7.8	5.7–7.2
c	3.6–7.2	5.4–17.3	5.0–6.1	5.5	9.0	12.7	3.7–4.1	4.4–4.5	4.2	5.9	3.9–7.5	5.1–10.3	4.2–5.3	7.2	9.7	9.8–10.4	9.8–14.4	9.4–17.7	3.2–4.2	3.4–4.5	4.5	6.7	4.0–6.0	4.7–8.8	4.9–6.0	7.6–8.8	10.0–10.6	14.0–20.8
Stl	1–7	3–6	n/a	n/a	5	5	8–9	8–9	n/a	n/a	8–9	8–9	n/a	n/a	n/a	n/a	n/a	n/a	n/a	n/a	n/a	n/a	7–8	6–8	n/a	n/a	n/a	n/a
Stw	1–4	2–5	n/a	n/a	n/a	n/a	3	2–3	n/a	n/a	3	3–4	n/a	n/a	n/a	n/a	n/a	n/a	n/a	n/a	n/a	n/a	n/a	n/a	n/a	n/a	n/a	n/a
Bdcs	4–12	6–14	n/a	n/a	6–7	6–7	8	8–9	n/a	n/a	n/a	n/a	10–11	11	n/a	n/a	n/a	n/a	n/a	n/a	n/a	n/a	8–10	6–7	7–9	6–9	7–8	7–9
Csla	0.6–0.8	0.3–0.3	n/a	n/a	Absent	Absent	n/a	n/a	n/a	n/a	1–2	1–2	1	1	n/a	n/a	n/a	n/a	n/a	n/a	n/a	n/a	n/a	n/a	0.4–0.5	0.4–0.5	1.4–1.5	1.5
Aw	1.3–4.2	2.3–3.6	n/a	n/a	2	2	3–4	3	n/a	n/a	3	3–4	n/a	n/a	n/a	n/a	n/a	n/a	n/a	n/a	n/a	n/a	3–5	3–5	2.8–4.0	2.8–3.0	4	3.5–4.0
Al	1.3–4.2	2.3–3.6	n/a	n/a	2	2	3–4	3	n/a	n/a	n/a	n/a	n/a	n/a	n/a	n/a	n/a	n/a	n/a	n/a	n/a	n/a	3–5	3–5	3.0–4.0	3.5–4.0	3.5–4	3.5–4.0
DaA	10.8–20.0	13.8–17.8	n/a	n/a	3–8	3–8	9–10	9	n/a	n/a	10–14	10–13	17–18	18	n/a	n/a	n/a	n/a	n/a	n/a	n/a	n/a	7.8–11.7	7.8–11.7	10.0–12.0	10.0–11.0	8–9	8–9
A%	10.0–31.4	13.0–26.2	n/a	n/a	10.7	1–8	23.0–30.6	23.6–29.8	n/a	n/a	n/a	n/a	n/a	n/a	n/a	n/a	n/a	n/a	n/a	n/a	n/a	n/a	n/a	n/a	21.5–30.8	23.1–27.3	30.8–36.4	30.8–33
BdA	8.8–19.5	10.9–17.3	n/a	n/a	n/a	n/a	11–12	11–12	n/a	n/a	n/a	n/a	n/a	n/a	n/a	n/a	n/a	n/a	n/a	n/a	n/a	n/a	n/a	n/a	10–12	10–11	11–13	11–13
Ol	2.1–3.8	2.3–5.3	n/a	n/a	n/a	n/a	n/a	n/a	n/a	n/a	n/a	n/a	n/a	n/a	n/a	n/a	n/a	n/a	n/a	n/a	n/a	n/a	absent	absent	2	1.5–2	3	3
Ow	1.9–3.5	1.4–3.6	n/a	n/a	n/a	n/a	n/a	n/a	n/a	n/a	n/a	n/a	n/a	n/a	n/a	n/a	n/a	n/a	n/a	n/a	n/a	n/a	absent	absent	2	1.5–2	3	3
Dao	17.6–38.7	25.4–33.4	n/a	n/a	27–37	27–37	35–44	40–49	n/a	n/a	43–49	49–59	42–45	45	n/a	n/a	n/a	n/a	n/a	n/a	n/a	n/a	absent	absent	20–36	21–26	30–45	30–38
Bdo	13.5–20.1	1.2–25.4	n/a	n/a	n/a	n/a	17–20	17–18	n/a	n/a	n/a	n/a	n/a	n/a	n/a	n/a	n/a	n/a	n/a	n/a	n/a	n/a	absent	absent	14–18	13–15	17	14–20
Bdph	15.3–29.8	19.3–35.3	n/a	n/a	n/a	n/a	21–24	21–24	n/a	n/a	n/a	n/a	n/a	n/a	n/a	n/a	n/a	n/a	n/a	n/a	n/a	n/a	n/a	n/a	19–23	19–20	130–136	122–152
Daph	102–168	103–181	n/a	n/a	97–130	97–130	108–123	113–118	n/a	n/a	n/a	n/a	n/a	n/a	n/a	n/a	n/a	n/a	n/a	n/a	n/a	n/a	85–115	80–95	120–122	109–126	130–136	122–152
Mbd	17–35	23–42	22	17	19	22	26–33	25–32	35	35	40	34	28	24	23	17	31	28	19	18	28	24	20–25	15–17	20–26	20–21	27	28
Abd	15–28	17–34	n/a	n/a	15–17	15–17	14–16	15–17	n/a	n/a	23–24	22–26	n/a	n/a	n/a	n/a	n/a	n/a	n/a	n/a	n/a	n/a	15–16	12–18	15–21	16–19	18–24	20–23
Tl	88–210	53–123	n/a	n/a	65–98	34–60	188–209	164–186	n/a	n/a	233–268	152–190	154–196	116	n/a	n/a	n/a	n/a	n/a	n/a	n/a	n/a	110–175	85–110	138–147	77–87	95–100	53–54
Vbd	19–31	n/a	n/a	n/a	n/a	n/a	24–28	n/a	n/a	n/a	n/a	n/a	n/a	n/a	n/a	n/a	n/a	n/a	n/a	n/a	n/a	n/a	20–22	n/a	n/a	n/a	n/a	n/a
VtA	126–285	n/a	n/a	n/a	n/a	n/a	n/a	n/a	n/a	n/a	n/a	n/a	168–175	n/a	n/a	n/a	n/a	n/a	n/a	n/a	n/a	n/a	50–165	n/a	n/a	n/a	n/a	n/a
V%	50–62	n/a	58–65	n/a	64–65	n/a	52–54	n/a	54	n/a	53–64	n/a	56–59	n/a	65	n/a	62–68	n/a	49–54	n/a	55–56	n/a	55–62	n/a	57–61	n/a	69–74	n/a
B	n/a	22–55	n/a	n/a	n/a	n/a	n/a	n/a	n/a	n/a	n/a	n/a	n/a	n/a	n/a	n/a	n/a	n/a	n/a	n/a	n/a	n/a	n/a	n/a	n/a	38.4	n/a	50
Pb	n/a	2 + 2	n/a	1 + 2 + 2	n/a	2 + 2	n/a	2 + 2	n/a	1 + 2	n/a	2 + 2	n/a	1 + 2 + 1 + 1	n/a	1 + 2	n/a	1 + 2	n/a	2 + 2	n/a	1 + 1 + 1 + 1 + 1	n/a	2 + 2	n/a	2 + 2	n/a	1 + 2
Ppc	≥1	1	n/a	0	n/a	n/a	n/a	n/a	n/a	1	n/a	0	n/a	n/a	n/a	n/a	n/a	n/a	n/a	n/a	n/a	n/a	n/a	n/a	n/a	2	n/a	n/a
Pcd	≥3	≥3	n/a	2	n/a	2	4 + 2	2	n/a	n/a	n/a	2	n/a	0														
S	n/a	57–90	n/a	62–70	n/a	31–38	n/a	45–50	n/a	45–50	n/a	48–78	n/a	46	n/a	27	n/a	35–41	n/a	27–33	n/a	45	n/a	46	n/a	60–67	n/a	33–39
Lfilt	30–139	27–87	n/a	n/a	n/a	n/a	n/a	n/a	n/a	n/a	n/a	n/a	n/a	n/a	n/a	n/a	n/a	n/a	n/a	n/a	n/a	n/a	n/a	n/a	n/a	51–64	20–26	18–26
Ltip	4–12	4–8	n/a	n/a	n/a	n/a	n/a	n/a	n/a	n/a	n/a	n/a	n/a	n/a	n/a	n/a	n/a	n/a	n/a	n/a	n/a	n/a	n/a	n/a	n/a	6–8	4–5	4–6

a Indicates measurements from scanning electron microscopy images.

### Differential species diagnosis

Morphologically, nematodes from GSL are most similar to five species that exhibit four pairs of bursal papillae in a (2 + 2) pattern: *D. delyi* Andrássy, 1958, *D. brucei* Hopper, 1970, *D. tehuelchus* Pastor de Ward and Lo Russo, 2009, *D. rushikondai* Sufyan et al., 2014, and *D. contortus* Chen et al., 2023. *D. woaabi* sp. nov. are distinct from *D. brucei* by an abrupt transition of the tail from conical to filiform, rather than the elongated conoid anterior and short cylindrical posterior tails of *D. brucei*. *D. woaabi* sp. nov. also exhibit a shorter gubernaculum (mean length:17.853 µm) than *D. brucei* (23–28 µm), and longer spicules (57.1–89.5 µm in *D. woaabi* vs. 31–38 µm in *D. brucei*). *D. woaabi* sp. nov. are distinct from *D. delyi*, *D. rushikonda*i, and *D. contortus* by having a relatively short male tail <5 abd, and thus relatively large de Man’s ratio *c* of 10.0. *D. woaabi* sp. nov. are distinct from *D. tehuelchus* by the number of pre-cloacal papillae within bursa (1 vs. 2), absence of mid-ventral post-cloacal papillae within bursa, and number and pattern of tail papillae after bursa (see updated Species Key for Genus *Diplolaimelloides* and Fig. 11A).

*D. woaabi* sp. nov. is further distinguished from *D. rushikonda* by the presence of ocelli (absent in *D. rushikonda*). *D. woaabi* sp. nov. is further distinguished from *D. delyi* by having a well cuticularized funnel-shaped gubernaculum vs. “simple thickening of posterior rectal lining” in *D. delyi*. They also exhibit filiform tail papillae that are absent in descriptions of *D. delyi*. *D. woaabi* are distinguished from several *Diplolaimelloides* species by a relatively short stoma length, long distance from anterior to amphid, and short distance from anterior to ocelli (Table 2).

Finally, we note three morphological features present in *D. woaabi* sp. nov. that appear to be unique. First, they exhibit “mid-tail” papillae in an offset, paired configuration (Figs. 6B–D; 11A). Second, the spicule in *D. woaabi* sp. nov. have extensions below the tip that are presumably used for latching to the female during copulation (i.e., the “halberd,” Figs. 6A; 11D). Third, they are the first species to be reported in the genus with fused lips, and this trait may warrant an update of the genus diagnosis in the future (Figs. 7B–F). We note that while the fused lips were readily apparent in SEM (Figs. 7C–F), they would be more difficult to observe with light microscopy alone. Moreover, the extensions on the spicule are more clearly seen when they are outside of the body; although they could be observed internally as well in a folded-up position (Fig. 11).

#### Etymology

The field sites from which we collected GSL nematodes belong to the ancestral tribal land of the Northwestern Band of the Shoshone Nation (NWBSN). *woaabi* was chosen by local NWBSN leaders, which is derived from *wó aabi*, the Shoshone word for “worm.”

#### Type locality and habitat

**Figure 12:**
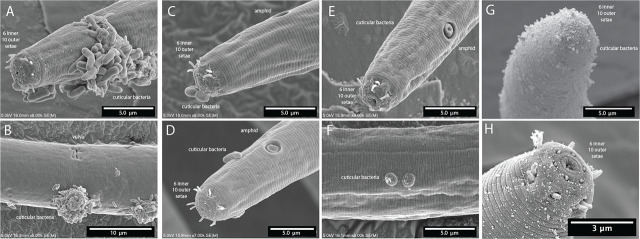
Scanning electron microscopy of body ornamentation or adherence of other organisms and/or substances on *Diplolaimelloides woaabi* sp. nov. that are (A–D) rod-like, (E,F) wart-like, or (G,H) fuzz-like.

*D. woaabi* sp. nov. type specimens were found in microbialite with a mean water salinity of 115 ppt, in a bay located 4,200 ft above sea level. The cuticular adherence of other organisms and substrates was apparent on these nematodes (Fig. 12).

#### Type designation and deposition

Several slides with individual specimen and a glass jar with approximately 30 specimens deposited at the Natural History Museum of Utah, UMNH.A.2025.7, Col. J. Jung, 7 October 2024, Great Salt Lake.

### Molecular analyses and phylogeny

SSU phylogenetic analyses using primers SSU18A/RH5401 and SSU26R/RH5402 are consistent with recent 18S-based phylogenetic trees of Monhysteridae (Westerman et al., 2022). *D. woaabi* specimens are in a well-supported clade containing *Diplolaimelloides delyi*, *Diplolaimelloides meyli*, an uncharacterized *Diplolaimelloides* species, *Diplolaimella dievengatensis* and *G. scyllae* (Fig. 3). This tree supports the placement of GSL nematodes in the *Diplolaimelloides* genus with *D. delyi* as its closest conger.

## Discussion

**Figure 13:**
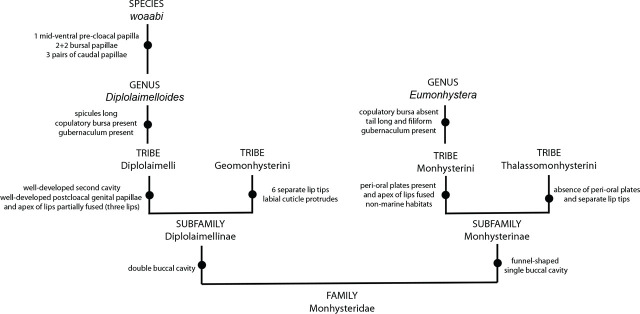
Differential diagnosis to identify *Diplolaimelloides woaabi* sp. nov.

This study adds a new species to the genus *Diplolaimelloides* Meyl, 1954 (Fig. 13). An overview of the genus indicates groupings, and potentially monophyly, based on the number and pattern of bursal papillae. Under this framework, *D. woaabi* sp. nov. falls within the “(2 + 2)” group alongside *D. delyi*, *D. brucei*, *D. tehuelchu*s, *D. rushikondai*, and *D. contortus*, representing 43% of described species within the genus. Another large group consists of species with a “1 + 2” bursal papillae, representing 36% of the genus. The locations from where the described species have been recovered lend modest support to this hypothesis: the “1 + 2” group are all Eurasian except for *D. warwicki* (Patagonia, Argentina) (Chen et al., 2023). However, the “(2 + 2)” group is more widespread, including species from North and South America, southern India, Thailand and China (Chen et al., 2023). Sampling and descriptions of more *Diplolaimelloides* specimens will provide a fuller picture of their distribution, and whether any biogeographic patterns can be found.

**Figure 14:**
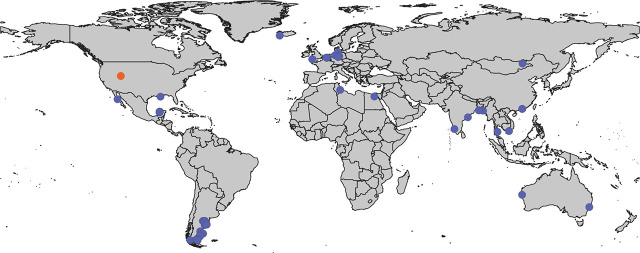
Global map of *Diplolaimelloides*. GIS map of documented *Diplolaimelloides* recovered from the wild (*n* = 63), representing 14 species. Where possible, exact GPS coordinates are used directly from source material including species-description manuscripts, images of notebooks, and the GBIF webserver. In a few cases, approximate coordinates are estimated from geographical description. *D. woaabi* sp. nov. depicted in orange. GBIF, Global Biodiversity Information Facility; GIS, Geographic Information Systems.

A more obvious pattern can be observed regarding habitat: most species of *Diplolaimelloides* have been isolated from coastal areas (Fig. 14). Yet, *D. palustris* (Mongolia) and *D. woaabi* sp. nov. (Utah) are >800 Km from the nearest coast. How did coastal nematodes colonize such inland locations? Approximately 100 million years ago, there was an inland sea that bifurcated modern-day North America into two land masses (Stanley, 2014). Intriguingly, the current boundaries of GSL were on the western edge of this sea. Assessing the systematics of *Diplolaimelloides* in the context of past geography may shed light on their current distribution – and the repeated environmental transitions that have occurred in Monhysteridae (Holterman et al., 2019).

In some SEM images we noticed microbes adhered to the cuticle of *D. woaabi* (Fig. 12). The cuticular adherence of other organisms and substrates is a common occurrence in nematodes (Das, et al., 2024; Decraemer et al., 2013). The epicuticle lies on the external surface of the nematode and appears to act as a hydrophobic barrier to diffusion. The epidermis, S-E system, and gland cells synthesize a glycoprotein-rich, negatively charged surface coat or glycocalyx, which overlies the epicuticle (Sharon et al., 2002). In natural environments, it may facilitate locomotion by lubrication (Bird et al., 1988) and protect the nematode from microbial predators, parasites, and pathogens. In *C. elegan*s, genes have been identified that alter the surface coat in such a way as to affect the adherence of a bacterium biofilm and thus affect bacterial pathogenesis (Darby et al., 2007). In a subfamily of marine-sediment inhabiting nematodes, the Stilbonematinae, the body cuticle is covered by ectosymbiotic sulfur-oxidizing bacteria enveloped in a mucus layer produced by a system of glandular sensory organs beneath the cuticle. GSL nematode-associated bacteria have recently been identified (Jung et al., 2024a), but the authors did not distinguish between bacteria on the surface of nematodes and bacteria within (presumably consumed by) these nematodes. Moreover, the nature and function of such ornamentation, that is, whether these are symbiotic cuticular bacteria or pathogenic (Davies et al., 2008), or if they facilitate locomotion as in other nematodes (e.g., Epsilonematidae, Criconematidae (Decraemer et al., 2013), is unknown.

**Revised key to species of the genus Diplolaimelloides**

1. Bursal papillae in pattern (2 + 2)……………………………………………………….…......2
  Bursal papillae in other patterns………………………………………………………………………….7
2. Male tail conical at anterior, filamentous in posterior part, filiform part more than half of tail length……………………………………………………………………………….……………….3
  Male tail elongate-conoid, 2.0–3.5 abd, posterior two-fifths cylindroid……………………………………… ………………………………………….*D. bruciei* Hopper, 1970
3. Male tail length (distance from anus/cloacal opening to posterior end) less than 5 abd…………………… ………………………………………………………………………………………. 4
  Male tail length (distance from anus/cloacal opening to posterior end) more than 5 abd………………… ………………………………………………………………………………..……… 5
4. Single pre-cloacal papilla within bursa, offset pair of dorso-lateral mid-tail papillae, spicules with extensions ……………………………………………………………*D. woaab*i nov. sp.
  A pair of pre-cloacal papillae and a pair of post-cloacal papillae within bursa; four pairs of midventral and sublateral papilla and seven unpaired midventral papillae on tail after bursa …………….…………………………………………....*D. tehuelchus* de Ward and Russo, 2009
5. Ocelli absent………………………………………………..*D. rushikondai* Sufyan et al., 2014
  Ocelli present….................................................................................................................................6
6. Spicules distinctly cephalate and bearing light transverse striations; spicule tip heavily sclerotized on ventral side but most of ventral side of blade unsclerotized and twisted outward, thin gubernaculum…………… …………..………………………*D. delyi* Andrássy, 1958 (Timm, 1966)
  Spicules twisted and curved at the middle portion, which are developed into a lateral plate connecting the proximal and distal ends of the spicule………..*D. contortus* Chen et al., 2021
7. Male without caudal papilla…………………………………………………………………8
  Male with caudal papillae…………………………………………………………….……….9
8. Bursal papillae in pattern (1 + 1 + 1 + 1 + 1)………………………… *D. palustris* Tsaliolikhin, 1985
  Bursal papillae in pattern (1 + 2 + 1 + 1)……………………….*D. elegan*s Gagarin and Thanh, 2008
9. With 3 papillae in bursa………………………………………………………………………10
  With 5 papillae in bursa…………………………………………………….……*D. altherri* Meyl, 1954
10. Male tail relatively long, length 14 abd………….………………………*D. oschei* Meyl, 1954
  Male tail relatively short, length less than 10 abd……………………………………………11
11. With one male tail papilla……………………………………………………………….….…12
  With more male tail papillae………………………………………………………….............13
12. Spicules shorter than 30 µm……………………………….*D. islandicus* (De Coninick, 1943)
  Spicules longer than 30 µm………………………………..……*D. deconincki* (Gerlach 1951)
13. Spicules angular and long gubernaculum……………..*D. warwicki* de Ward and Russo, 2009
  Spicules arcuate and short gubernaculum…………………………........*D. meyli* Timm, 1961

Finally, the unique habitat of *D. woaabi* sp. nov. suggests both physiological and ecological adaptation. A potential benefit of adapting to extreme environments is the avoidance of predators or competition (dos Santos et al., 2009; dos Santos and Moens, 2011). Consistent with this idea, we previously observed a complex community of nematodes in a freshwater river that flows into GSL. This biodiversity transitions abruptly to a single family as the river empties into the hypersaline portion of the lake (Jung et al., 2024a). However, we note that a full survey of the lake has not yet been done. Moreover, it is currently unknown to what extent population structure exists within GSL, and/or the presence of cryptic species. More extensive sampling of the lake in the future will help to answer these questions, especially when paired to genomic and transcriptomic sequencing. Although the amount of DNA in single nematodes has posed a historical challenge for sequencing approaches, newer methods are emerging that lower the effective input requirements for library preparation (Lee et al., 2023; Wang et al., 2024). Implementing these methods with wild-isolates of *Diplolaimelloides* has great potential to update its systematics, and serve as a model system for the evolution of animals to extreme environments.
